# Usage of nivolumab and ipilimumab for recurrent or advanced malignant vaginal melanoma: a two-case series

**DOI:** 10.1007/s00795-023-00377-6

**Published:** 2024-01-30

**Authors:** Kota Konishi, Mamiko Okamoto, Ryuichi Tokumitsu, Mitsutake Yano, Kaei Nasu, Eiji Kobayashi

**Affiliations:** 1 Department of Obstetrics and Gynecology, Faculty of Medicine, Oita University, 1-1 Idaigaoka, Hasamamachi, Yufu, Oita 879-5593 Japan; 2 Division of Obstetrics and Gynecology, Support System for Community Medicine, Faculty of Medicine, Oita University, Oita, Japan

**Keywords:** Abscopal effect, Concurrent radiotherapy, Immune checkpoint inhibitor, Immune-related adverse event, Malignant vaginal melanoma, Neoadjuvant chemotherapy

## Abstract

Immune checkpoint inhibitors help treat malignant melanoma, but show limited use in treating malignant vaginal melanoma, an aggressive, rare gynecological malignancy. We identified two patients treated with ipilimumab and nivolumab for vaginal melanoma; both were immunonegative for programmed cell death-ligand 1 and wild-type *BRAF*. Case 1, a 56-year-old female who underwent radical surgery for stage 1 malignant vaginal melanoma, experienced recurrence 15 months postoperatively. She briefly responded to ipilimumab and nivolumab combination therapy before showing disease progression. Tumor shrinkage occurred with nivolumab and local radiotherapy and, 45 months postoperatively, she survives with the melanoma. Case 2, a 50-year-old female, presented with a 4-cm blackish polypoid vaginal tumor with metastatic pelvic lymph nodes. She received ipilimumab and nivolumab combination therapy for stage III unresectable malignant vaginal melanoma. The vaginal tumor shrank after the third course of treatment, and the lymphadenopathy disappeared. The patient underwent radical surgery and is currently disease-free, using nivolumab for maintenance therapy. Both patients had immune-related adverse events coinciding with periods of high therapeutic efficacy of immune checkpoint inhibitors. Neoadjuvant therapy with immune checkpoint inhibitors and radiotherapy for immune checkpoint inhibitor resensitization may effectively treat advanced or recurrent vaginal melanoma.

## Introduction

Malignant vaginal melanoma—aggressive and rare—is a gynecological malignancy that accounts for 3% of all vaginal cancers and 0.3% to 0.8% of all malignant melanomas [1]. Immune checkpoint inhibitors (ICIs) help treat malignant melanoma; however, their use in treating vaginal malignant melanoma is limited. In particular, whether ICIs should be used alone or in combination to treat vaginal malignant melanoma [2, 3], whether they are useful as adjuvants [4] or neoadjuvant chemotherapy (NAC) [5], and whether they have synergistic effects with radiotherapy [6] is controversial. Herein, we report two cases in which combination therapy with ipilimumab and nivolumab was used to treat advanced or recurrent vaginal melanoma. Our findings on the usefulness of NAC with ICI, resensitization to ICIs using radiation, and immune-related adverse events (irAEs) suggest new therapeutic strategies for treating malignant vaginal melanomas.

## Case reports

### Case 1

**Fig. 1:**
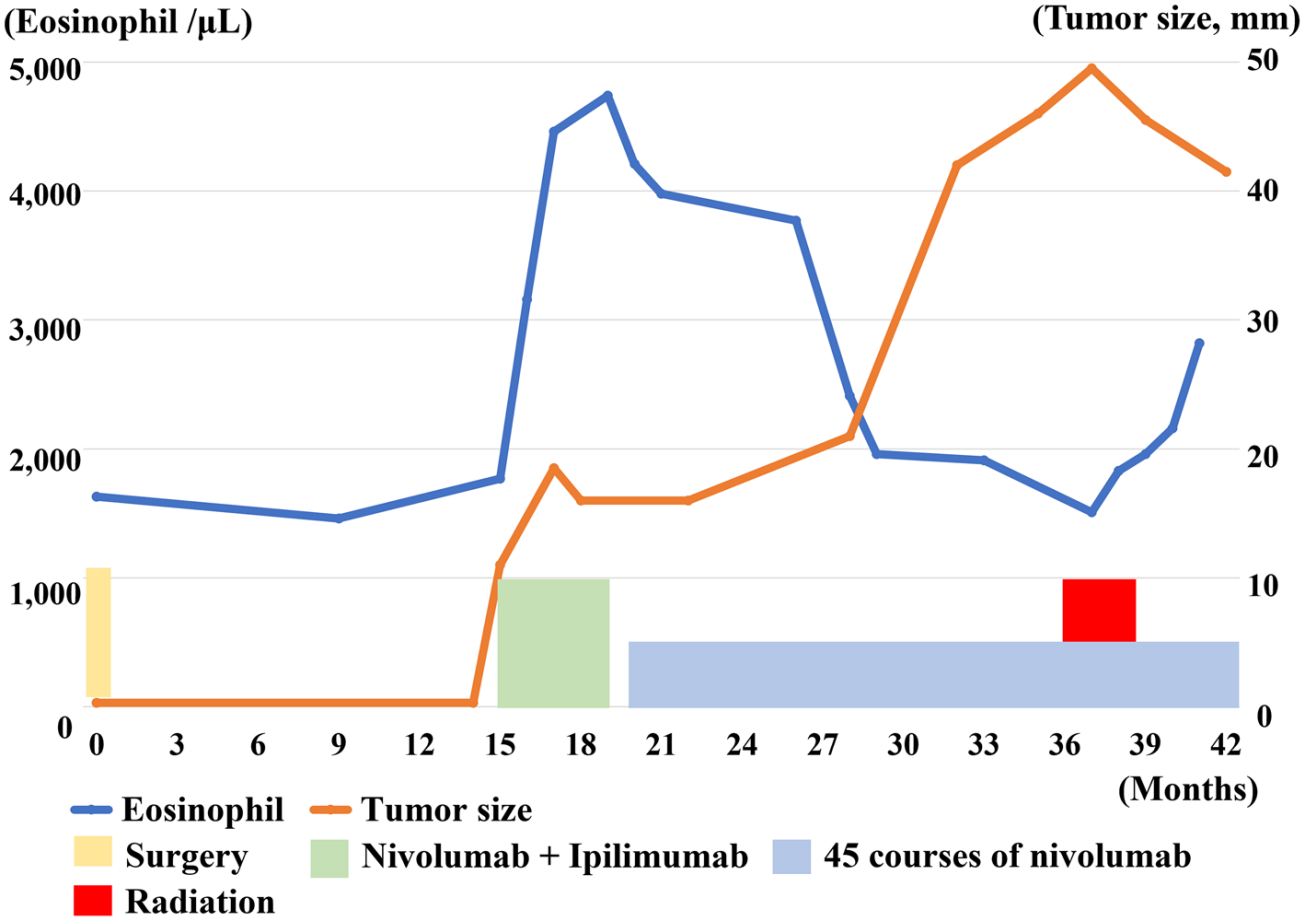
Clinical course of Case 1. Tumor suppression and irAE/eosinophilia by ICIs were inversely correlated. Local radiotherapy enhanced the tumor shrinkage and eosinophilia induced by ICIs

**Fig. 2:**
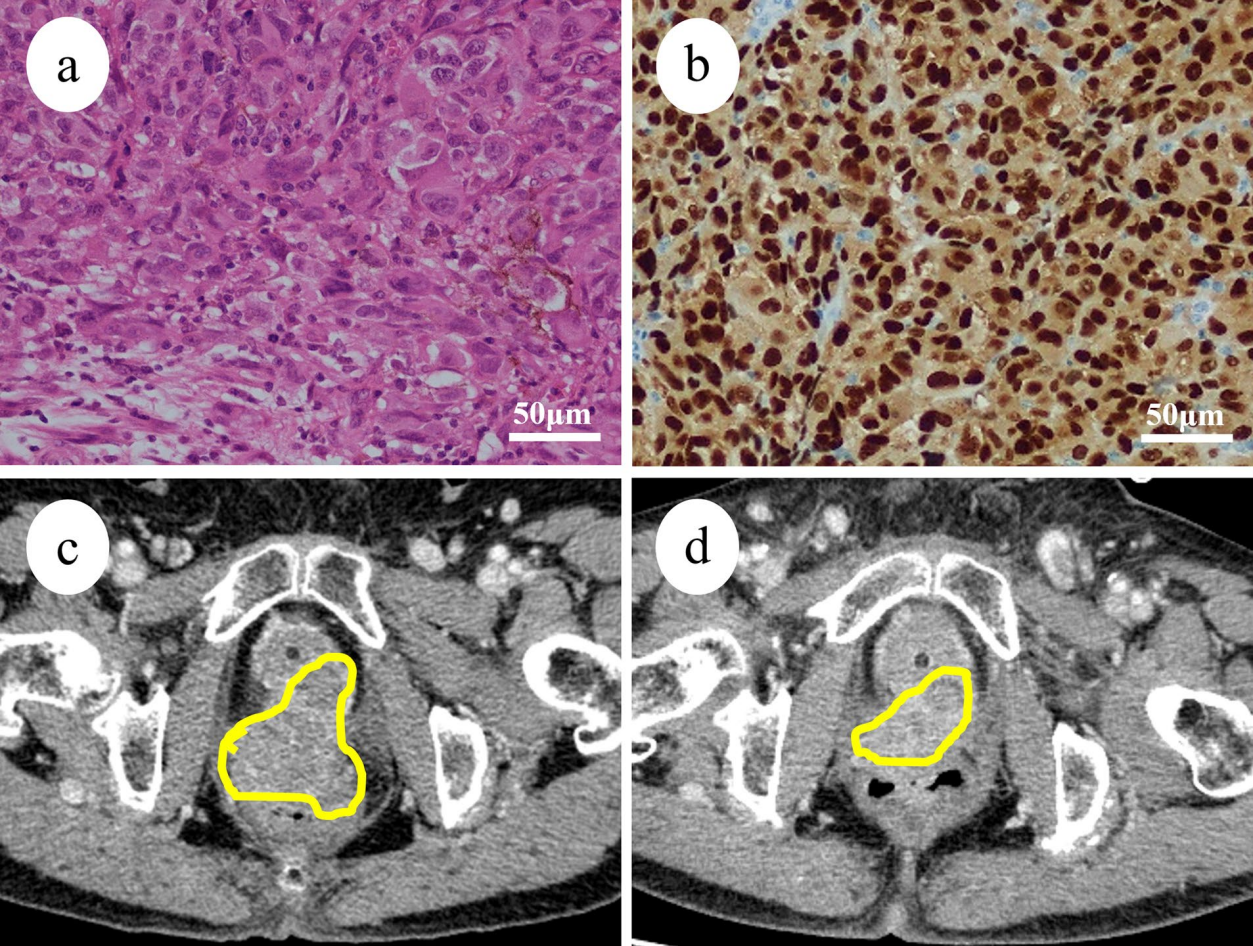
Clinical and pathological imaging of Case 1. **a** Histological examination revealed infiltrative growth of atypical cells with melanin production. **b** Immunohistochemically, the tumor cell is positive for SOX-10. Computed tomography examination **a** at the time of progression during nivolumab monotherapy (yellow line, tumor) and **b** after combination therapy with nivolumab and radiotherapy (yellow line, tumor)

A 56-year-old female, with a gravidity of two and parity of one, had a history of eosinophilic esophagitis. The clinical course is summarized in Fig. 1. The patient underwent extended stage I malignant vaginal melanoma surgery at our hospital. The tumor with melanin production was immunohistochemically positive for S-100, HMG-45, Melan-A, and SOX10 (Fig. 2a, b). *BRAF* was wild-type, and programmed death-ligand 1 immunohistochemical staining was negative. Adjuvant therapy was not administered. Contrast-enhanced magnetic resonance imaging (MRI) and contrast-enhanced computed tomography (CT) examinations, performed 1 year and 3 months after surgery, revealed a recurrent pelvic mass 1–2 cm in size. The patient began triweekly combination therapy of nivolumab 80 mg/body and ipilimumab 3 mg/kg. At this time, the percentage of eosinophils was 7.2%. After three courses of nivolumab-ipilimumab combination therapy, the patient presented with a mood disorder and diarrhea. Blood tests showed that the adrenocorticotropic hormone and cortisol levels were < 1.50 pg/mL and 3.02 μg/dL, respectively. The patient was diagnosed with grade 3 pituitary dysfunction as an irAE and underwent hormone replacement therapy. The percentage of eosinophils was 37.8% at this time. Contrast-enhanced CT revealed stable disease, and she underwent a fourth course of nivolumab-ipilimumab combination therapy. After that, her treatment was switched to nivolumab monotherapy every 2 weeks. After 24 courses of single-agent nivolumab, 240 mg/body biweekly, contrast-enhanced CT revealed an enlarged pelvic tumor (Fig. 2c). The percentage of eosinophils at this point was 17.0%. Gene panel testing revealed microsatellite stability, low tumor mutation burden, and genetic mutations in *SPEN* and *FGFR4*. Therefore, we decided to continue nivolumab monotherapy and add radiation therapy. The patient was treated with pelvic radiation at a dose of 45 Grey in 25 fractions. Subsequently, she continued nivolumab monotherapy and the tumor showed shrinkage (Fig. 2d). At this time, the percentage of eosinophils was 49.0%. Symptoms of eosinophilic esophagitis appeared but weakened without treatment. To date, the patient has undergone 49 doses of nivolumab monotherapy within the 3 years and 9 months since the initial surgery and has progression-free survival.

### Case 2

**Fig. 3:**
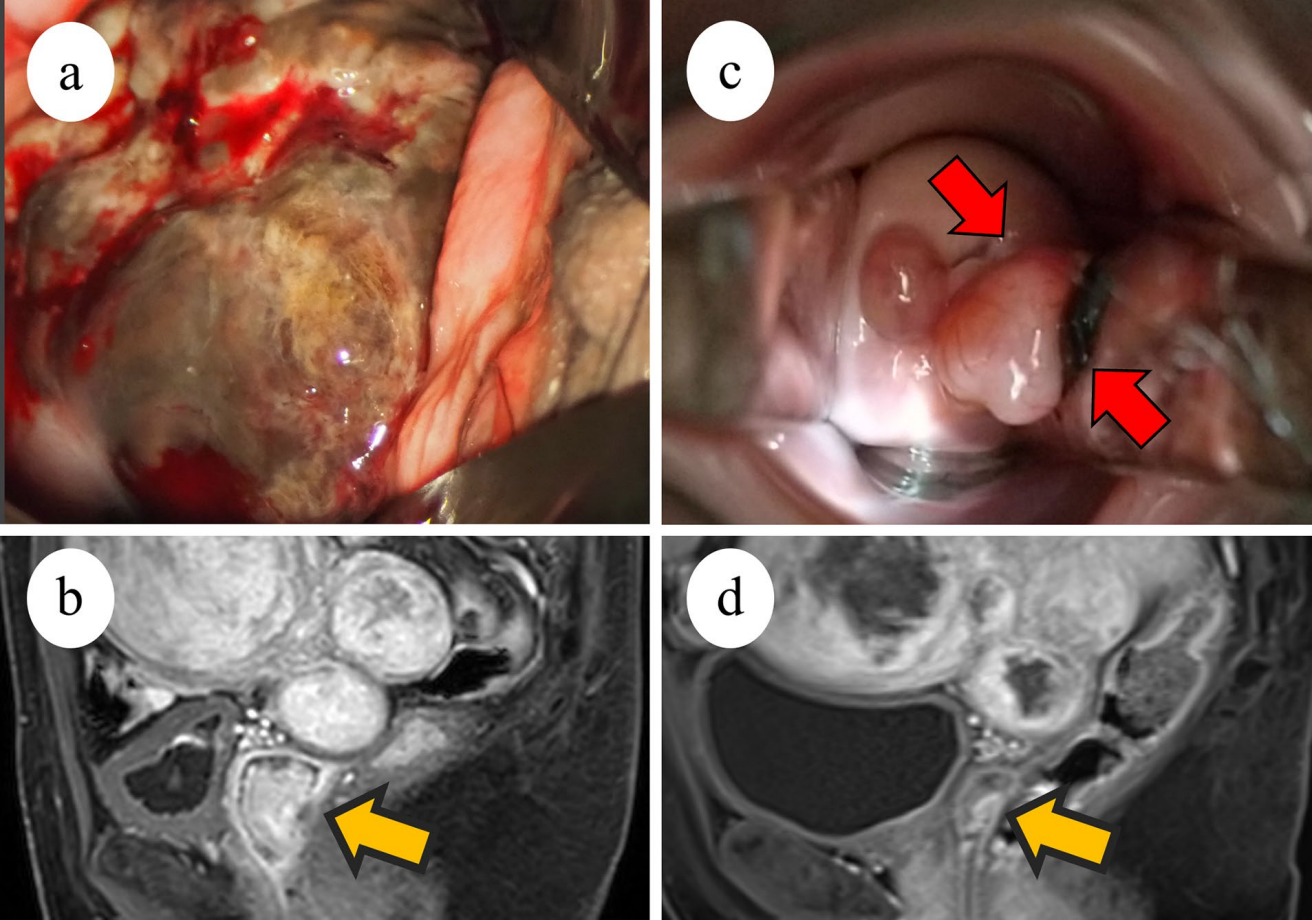
Clinical imaging of Case 2. **a** Colposcopy and **b** magnetic resonance imaging examination (yellow arrow, tumor) before combination therapy with ipilimumab and nivolumab. **c** Colposcopy (red arrows, tumor) and **d** magnetic resonance imaging (yellow arrow, tumor) after combination therapy with ipilimumab and nivolumab

**Fig. 4:**
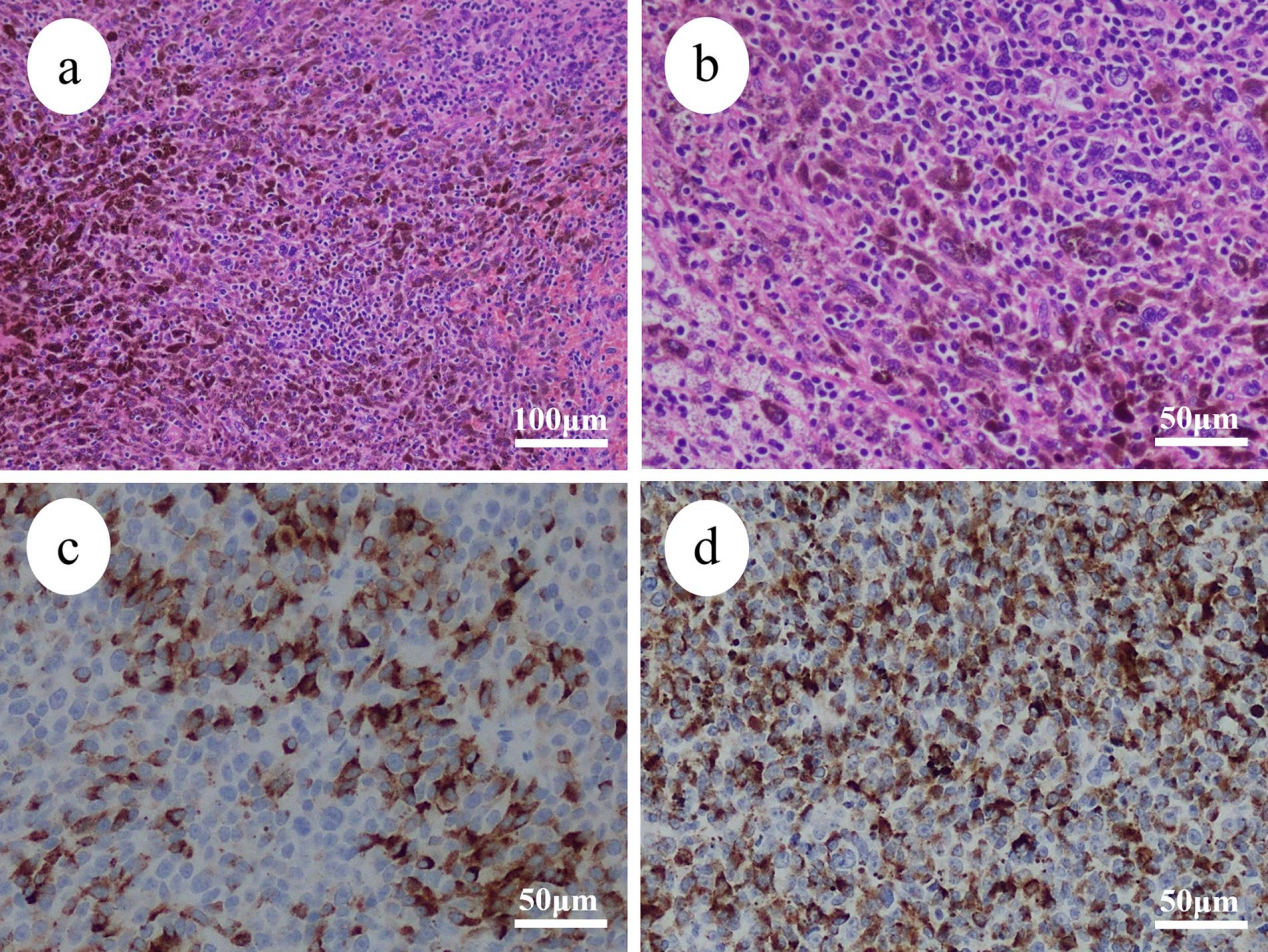
Pathological imaging of Case 2. **a** low power view; **b** high power view Histologically, the tumor showed infiltrative growth of atypical cells with melanin production. Immunohistochemically, the tumor cell is positive for **c** Melan-A and **d** HMG-45

A 50-year-old female with one gravidity and one parity visited a local doctor to examine a vaginal mass; through tumor biopsy, malignant melanoma was diagnosed. The patient visited our hospital for treatment and reported a history of duodenal ulcers and hypertension. Her grandfather had a history of gastric cancer. Internal examination revealed a 4 cm black protruding mass in the middle third of the left vaginal wall (Fig. 3a). Contrast-enhanced MRI revealed no extravaginal tumor invasion (Fig. 3b). Contrast-enhanced CT revealed left external iliac lymph node metastasis. Histological examination revealed infiltrative growth of atypical cells with melanin production (Fig. 4a, b). Immunohistochemical staining revealed that the tumor cells were positive for S-100, HMG-45, Melan-A, and SOX10 and negative for AE1/AE3 (Fig. 4c, d). The *BRAF* gene was wild-type, and programmed death-ligand 1 immunohistochemical staining was negative. The patient was diagnosed with International Federation of Gynecology and Obstetrics stage III (cT1, cN1, and cM0) malignant vaginal melanoma, deemed inoperable. The patient began triweekly combination therapy of nivolumab 1 mg/kg and ipilimumab 3 mg/kg. After three courses, she was diagnosed with an irAE of grade 3 autoimmune hepatitis, and blood tests showed an AST of 520.2 IU/K and an ALT of 764.3 IU/L. After treatment with steroids and the immunosuppressant mycophenolate mofetil, her liver dysfunction improved to grade 1. Three months after the initial treatment, contrast-enhanced MRI and internal examination revealed that the tumor had shrunk (Fig. 3c, d) and the left external iliac lymph node metastasis had disappeared. The tumor was considered operable, and the patient underwent a radical hysterectomy, bilateral adnexectomy, and pelvic lymph node dissection. The excised specimen had a 10 mm-sized tumor in the vagina, located mainly on the left wall, with a black surface. Histological and immunohistochemical findings of the resected specimen were similar to those of the previous biopsy. The resection margins were negative. No uterine, adnexal, or pelvic lymph node metastases were observed. The patient continued postoperative steroid therapy and completed treatment with the immunosuppressant mycophenolate mofetil. One month after surgery, nivolumab monotherapy (240 mg/body triweekly) was started as maintenance chemotherapy. Twelve months after the initial treatment, the patient had been administered eleven doses of nivolumab monotherapy and is alive and disease-free.

## Discussion

The present case series suggests the following three novelties regarding malignant vaginal melanoma: (1) radiation promotes resensitization to ICIs when a vaginal melanoma becomes resistant, (2) radiation reactivates both antitumor effects and side effects, and (3) ICI adjuvant therapy can render unresectable cases resectable.

Malignant vaginal melanomas treated using ICIs are summarized in Table 1 [7–20]. In addition to those described in the current report, 17 cases have been identified, with patients ranging in age from 40 to 85 years. Only one *BRAF* mutation was identified, demonstrating the superiority of ICIs over BRAF inhibitors in treating vaginal melanoma. The two cases of vaginal malignant melanoma we encountered were successfully treated with a combination therapy of ipilimumab and nivolumab. However, currently, no standard treatment for progression during ICI therapy is available. In Case 1, nivolumab and local radiation showed antitumor effects after resistance to nivolumab was demonstrated, indicating resensitization to the ICIs. Yin et al. reported a case of recurrent malignant vaginal melanoma in a patient whose sensitivity to toripalimab and lenvatinib was improved using local radiotherapy [11]. Similarly, Schonewolf et al. reported resensitization to pembrolizumab after local radiotherapy in a case of advanced vaginal melanoma [7]. Here, we report the first case of radiation-induced resensitization to nivolumab, a key drug for treating malignant melanoma. Recently, the enhancement effect of ICI induced by local radiotherapy is referred to the abscopal effect. [6, 21–23].

**Table 1:** Use of immune checkpoint inhibitors to treat vaginal melanoma

Author (year of publication)	Patient age, years	PD-L1	BRAF	ICI purpose	ICIs	CRT	Outcome	irAE	Reference number
The present Case 1	56	Negative	Wild	Main therapy for recurrence	Ipilimumab + Nivolumab, 4 Cy; Nivolumab maintenance	Yes	PR	pituitary dysfunction, grade 3; eosinophilic esophagitis	NA
The present Case 2	50	Negative	Wild	NAC for stage III melanoma	Ipilimumab + Nivolumab, 3 Cy; Nivolumab maintenance	No	CR	Immune-mediated hepatitis, grade 3	NA
Schonewolf CA (2022) #1	56	NA	Wild	NAC for localized melanoma	Ipilimumab + Nivolumab, 4 Cy; Nivolumab maintenance	Yes	CR	NA	(7)
Schonewolf CA (2022) #2	80	NA	Wild	Main therapy for stage IVB melanoma	Pembrolizumab	Yes	PR	NA	(7)
Tarhini AA (2022)	40 s	Positive	Wild	Main therapy for locally advanced melanoma	Ipilimumab + Nivolumab, 3 Cy	No	CR	Immune-mediated hepatitis, grade 3	(8)
Ishiguro A (2022)	70	NA	Wild	Main therapy for localized melanoma	Nivolumab	No	PR	NA	(9)
Walz D (2022)	84	NA	Wild	Main therapy for stage III melanoma	Ipilimumab + Nivolumab, 1 Cy	No	NA	NA	(10)
Yin P (2022)	55	NA	Mutant	Main therapy for recurrence	Tripalimab	Yes	PR	NA	(11)
Guo N (2021)	58	Positive	NA	AC for stage III melanoma	Nivolumab, 6 Cy	No	CR	NA	(12)
Lambert L (2021)	54	NA	NA	Main therapy for recurrence	Pembrolizumab, 4 Cy	No	PR	Polymorphous vitelliform maculopathy	(13)
Sezen D (2021)	73	NA	NA	Main therapy for stage IIICb melanoma	Ipilimumab + Nivolumab, 3 Cy; Nivolumab 18 Cy	Yes	CR	Diarrhea, urethritis	(14)
Norwood TG (2019)	54	NA	NA	Main therapy for recurrence	Ipilimumab + Nivolumab, 3 Cy; Nivolumab maintenance	No	PR	Rash (grade 3), headache, hyponatremia, hypophysitis, colitis	(15)
Komatsu-Fujii T (2019)	85	NA	Wild	Main therapy for recurrence	Nivolumab, 3 Cy; Pembrolizumab, 4 Cy; Ipilimumab 4 Cy	No	PD	NA	(16)
Raad RA (2017)	60	NA	NA	Main therapy for stage III melanoma	Ipilimumab	NA	NA	Pneumonia	(17)
Mesko S (2017)	70	NA	NA	AC for surgical margin-positive melanoma	Ipilimumab, 4 Cy	Yes	CR	Skin reaction	(18)
Chanal J (2016)	72	Negative	Wild	Main therapy for recurrence	Ipilimumab, 4 Cy; Pembrolizumab, 18 Cy	No	CR	NA	(19)
Sano T (2016)	70	NA	NA	Main therapy for stage IVB melanoma	Nivolumab, 3 Cy	No	NA	Pneumonia	(20)

*AC* adjuvant chemotherapy, *CR* complete response, *CRT* concurrent chemoradiotherapy with ICIs, *Cy* cycles, *ICI* immune checkpoint inhibitor, *irAE* immune-related adverse event, *NA* not available, *NAC* neoadjuvant chemotherapy, *PD* progressive disease, *PD*-*L1* programmed death ligand 1, *PR* partial response, *PD* progressive disease

The synergistic effects of ICI and irradiation have been observed in other mucosal malignant melanomas [21] and different cancer types [22, 23]. These findings suggest that adding radiotherapy, rather than changing the drug, can be effective for ICI-resistant vaginal melanoma. We encountered cases of serious irAEs coinciding with periods of high therapeutic efficacy of ICIs [24]: correlations between multisystem irAEs and improved patient survival have been shown [25]. Notably, in Case 1, aggravation of adverse events (elevation of eosinophils) was observed in parallel with radiotherapy-induced resensitization to ICIs. Mesko et al. reported severe cutaneous irAEs associated with ICI and radiotherapy [18], suggesting that radiotherapy activates the effect of ICIs and irAEs, and that the occurrence of irAEs may be a marker of good antitumor effects.

The efficacy of ICI neoadjuvant therapy for vaginal melanomas remains controversial. Tarhini et al. reported a complete response to ipilimumab and nivolumab as preoperative therapy for locally advanced vaginal melanoma; however, the patient did not undergo surgery [8]. Schonewolf et al. reported the use of ipilimumab and nivolumab as NAC, but ultimately added radiotherapy after surgery [7]. Case 2 was the first case in which pure NAC with ICI therapy were administered: combination therapy with ipilimumab and nivolumab caused an inoperable malignant vaginal melanoma with pelvic lymph node metastasis to became operable. The efficacy of combination therapy with ipilimumab and nivolumab as NAC has been reported for malignant cutaneous melanoma [5], while the prognoses of surgical and nonsurgical cases of malignant cervical melanoma are similar [26]. In malignant vaginal melanomas, NAC with ICIs should be proactively introduced to avoid excessive surgical invasiveness.

## Conclusion

Neoadjuvant therapy with ICIs and radiotherapy for ICI resensitization may effectively treat advanced or recurrent vaginal melanoma. Adverse events may correlate with favorable treatment effects; therefore, premature termination of ICIs due to adverse events should be avoided.

## Data Availability

The data that support the findings of this study are available from the corresponding author upon reasonable request.
